# Short‐term morbidity and mortality following surgical treatment of anal squamous cell carcinoma in Sweden – A national multicentre study

**DOI:** 10.1111/codi.70241

**Published:** 2025-10-03

**Authors:** Stephanie Jacobsen, Eva Angenete, Anders Johnsson, Marie‐Louise Lydrup, Per J. Nilsson, Pamela Buchwald

**Affiliations:** ^1^ Department of Surgery Skåne University Hospital, Malmö, Lund University Lund Sweden; ^2^ Department of Surgery, SSORG‐Scandinavian Surgical Outcomes Research Group Institute of Clinical Sciences, Sahlgrenska Academy, University of Gothenburg Gothenburg Sweden; ^3^ Region Västra Götaland, Sahlgrenska University Hospital, Department of Surgery Sahlgrenska University Hospital Gothenburg Sweden; ^4^ Department of Oncology Skåne University Hospital Lund Sweden; ^5^ Department of Pelvic Cancer Karolinska University Hospital Stockholm Sweden

**Keywords:** abdominoperineal excision, anal cancer, Myocutaneous flap reconstruction, pelvic exenteration, perineal wound, postoperative complications

## Abstract

**Introduction:**

Anal squamous cell carcinoma (ASCC) is primarily treated with chemoradiotherapy (CRT), but surgery can be curative following treatment failure. The treatment of ASCC was centralised in Sweden in 2017. This study aims to describe the characteristics and outcomes of surgery for ASCC nationally, focusing on short‐term morbidity and mortality.

**Materials and Methods:**

All patients with ASCC who underwent surgery from 2017 to 2021 in Sweden were retrospectively identified. Perioperative complications and mortality rates were recorded, and risk factors were analysed.

**Results:**

A total of 97 patients underwent 103 surgical procedures. The median age was 64 years (56–74), and 61% were women. Indications included recurrent tumours after CRT (43%), residual disease (34%), re‐recurrence following prior salvage surgery (8%), primary surgery due to CRT contraindication (8%) and post‐treatment sequelae (8%). Surgical procedures included 84 (82%) abdominoperineal excisions, 10 (10%) pelvic exenterations, 5 (5%) perineal excisions for local re‐recurrences and 4 (4%) lymphadenectomies.

Overall, 48% of patients experienced perineal morbidity, defined as perineal wound infection, dehiscence, necrosis or pelvic abscess. Major surgical complications (Clavien‐Dindo ≥ III) were reported in 22/103 (21%) of procedures. Most reoperations (9/16) were related to flap complications. Thirty‐ and 90‐day mortality rates were 0% and 1%, respectively.

**Conclusion:**

This national cohort study presents the largest recent series of surgically treated ASCC patients in Sweden. Surgery caused substantial short‐term morbidity, particularly perineal morbidity, while short‐term mortality remained low. Although no significant risk factors for major complications were identified, these findings highlight the need for improved perineal wound management and further evaluation of long‐term outcomes.

**What does this paper add to the literature?**

This first nationwide evaluation of surgical outcomes for ASCC in Sweden highlights significant short‐term morbidity and underscores the need for improved perineal wound management, supporting future optimisation of surgical and reconstructive strategies in specialised clinical centres.

## INTRODUCTION

Anal squamous cell carcinoma (ASCC) is a rare malignancy strongly associated with high‐risk types of human papilloma virus (HPV) [1]. Other risk factors include immunosuppression, history of cervical dysplasia and smoking [2, 3]. Although CRT is the primary treatment option for most patients [4, 5, 6], surgery sometimes plays an important role in the management of ASCC patients. For early (T1N0) perianal tumours, local excision may suffice, and for patients with either a residual or recurrent tumour, salvage surgery is indicated. In addition, a small proportion of patients may have contraindications to CRT and are therefore candidates for primary surgical treatment. Finally, some patients may have refractory symptoms after CRT leading to surgery [7]. Abdominoperineal excision (APE) is performed in most patients, often requiring wide lateral skin margins and en bloc resection of adjacent viscera [8]. Extralevator APE (ELAPE) is commonly indicated to achieve negative resection margins, as the tumour often involves the pelvic floor [9, 10, 11]. Up to 70% of women undergo an extended resection including the posterior vaginal wall, and synchronous hysterectomy is recommended for women with HPV‐associated disease [7]. Pelvic exenterations after CRT for ASCC are associated with higher rates of major complications (41–61%) compared to exenterations for other pelvic malignancies [12]. Pelvic floor reconstruction with myocutaneous flaps promotes wound healing in previously irradiated tissue and decreases the risk of surgical‐site complications, making it a preferred method over primary wound closure [13, 14]. However, no consensus exists on the optimal approach to perineal reconstruction [11, 15, 16, 17].

The aims of this nationwide retrospective cohort study were to describe surgical approaches and the short‐term morbidity and mortality after surgical treatment for ASCC in Sweden between 2017 and 2021.

## MATERIALS AND METHODS

### Patients

Surgically treated ASCC patients in Sweden from January 2017 to December 2021 were retrospectively identified through local theatre lists at participating centres, encompassing all eligible patients nationally. Up until 2017, salvage surgery was performed at several different referral hospitals in Sweden. The centralisation of ASCC surgery began in 2017, designating Sahlgrenska University Hospital in Gothenburg and Skåne University Hospital in Malmö as the two national centres. During the implementation period, patients also underwent surgical treatment at Ersta Hospital and Karolinska University Hospital in Stockholm. All patients undergoing surgical treatment for ASCC irrespective of indication were included, that is patients with recurrent or residual ASCC, patients with locoregional recurrence after salvage surgery as well as patients receiving primary surgical treatment due to previous irradiation for other pelvic malignancy. In addition, patients undergoing surgical treatment due to radiation sequelae (i.e. fistulas, chronic wounds, intolerable perineal pain) were included. All perineal reconstructions were performed by plastic surgeons in collaboration with the colorectal surgical teams at the respective centres. All patients were discussed at a national multidisciplinary conference to assess surgical indication and determine appropriate treatment.

This retrospective observational cohort study was approved by the Swedish Ethical Review Authority (Dnr 2020–06085).

### Definitions

Residual or recurrent ASCC was diagnosed by biopsy or radiological suspicion, with residual disease diagnosed within 6 months of completing CRT and recurrence after 6 months. Re‐recurrence was defined as locoregional relapse after prior salvage surgery.

Total pelvic exenteration was defined as APE with cystectomy and resection of the internal reproductive organs. Posterior exenteration involved APE with hysterectomy (± posterior vaginal wall), sparing the bladder. Anterior exenteration referred to cystectomy with reproductive organ resection. Local excision was defined as wide tumour removal after re‐recurrence, without anorectal excision.

### Data extraction

The Swedish anal cancer registry, initiated in 2015 and with a coverage of >95% of all ASCC cases nationwide, was used to extract data on clinical staging, tumour characteristics and oncological treatment [18]. Electronic patient charts were retrospectively reviewed for surgical data not obtainable from the national registry. Data on patient and treatment characteristics included indication for surgery, surgical approach, extent of resection and pelvic floor reconstruction. All 30‐day postoperative complications requiring medical and/or surgical intervention were recorded and classified according to the Clavien‐Dindo system (grades I–V), based on the invasiveness of the required intervention [19], with emphasis on major complications defined as a grade ≥ III.

### Statistical analysis

Descriptive statistics were used to present data. Continuous data were reported according to distribution as the median with interquartile range. Categorical data were presented as numbers with percentages.

A Directed Acyclic Graph was constructed to identify potential confounders and variables directly influencing postoperative outcomes, thereby guiding the selection of covariates for inclusion in the logistic regression analysis used to identify potential risk factors for major complications (Clavien‐Dindo ≥ III) following surgery (Figure S1). Variables included in the regression analysis were age at surgery, sex, CRT before surgery (yes/no), ASA physical status classification score, clinical TNM stage and tumour location (perianal/anal canal/rectum). Patients undergoing primary surgical treatment due to previous irradiation or salvage surgery with APE/extended APE as surgical procedures were included in the logistic regression analysis. Patients with missing data for a specific variable were excluded from the corresponding analysis. Analyses were performed using IBM SPSS Statistics version 30.

## RESULTS

### Patient and surgical characteristics

A total of 97 patients treated between 2017 and 2021 were identified from local theatre lists at the hospitals in Malmö, Gothenburg and Stockholm. During the study period, four patients underwent at least one reoperation. In total, 103 surgical procedures were performed.

Patient characteristics are summarised in Table 1. P16‐positivity (surrogate marker for HPV), determined by immunohistochemistry, was confirmed in 69/97 (71%) patients. Fifty of 59 (85%) female patients and 19/38 (50%) of male patients were p16‐positive. Treatment adhered to the Swedish national guidelines for ASCC radiation therapy [18]. CRT was given mainly as 5‐fluouracil or capecitabine, both with mitomycin C. Radiotherapy was typically delivered in 27 fractions. Median doses were 57.5 Gy (57.5–58.0 Gy) to the primary tumour, 50.5 Gy (50.5–54.0 Gy) to lymph node metastases and 41.6 Gy (41.6–56.0 Gy) to elective lymph nodes.

**TABLE 1:** Patient demographics and preoperative variables including tumour characteristics before oncological treatment. Data are available for all included patients (*n* = 97) unless otherwise specified.

Characteristic	*n* (%)
Age, median (IQR)	64 (56–74)
Sex ratio, F: M	3:2
Female	59 (61)
Male	38 (39)
ASA physical status classification	
1	7 (7)
2	57 (59)
3	33 (34)
Body mass index (kg/m^2^), median (IQR)	24.4 (21.4–27.3)
Active tobacco use	21 (22)
P16 status^a^	
Positive	69 (71)
Negative	26 (27)
Not reported	2 (2)
Clinical T stage	
cT1	3 (3)
cT2	24 (25)
cT3	34 (35)
cT4	23 (24)
cTx	4 (4)
Not reported	9 (9)
Clinical *N* stage	
cN0	37 (38)
cN1	7 (7)
cN2	11 (11)
cN3	26 (27)
Not reported	16 (16)
Clinical M stage	
cM0	86 (89)
cM1	3 (3)
Not reported	8 (8)
Tumour location	
Perianal (margin)	10 (10)
Anal canal	32 (33)
Perianal + anal canal	27 (28)
Rectum	3 (3)
Rectum + anal canal	11 (11)
Perianal + anal canal + rectum	7 (7)
Not reported	7 (7)
Oncological treatment	
Primary tumour, total Gy	57.5 (57.5–58)
Elective lymph nodes, total Gy	41.6 (41.6–46.0)
Lymph node metastases, total Gy	50.5 (50.4–54.0)
Chemotherapy given	82 (85)

Abbreviations: ASA, American Society of Anaesthesiologists physical status classification system; IQR, interquartile range.

^a^ Surrogate marker for HPV‐status.

Surgical data are presented in Table 2. The most common indication for surgery was recurrent ASCC (43%), followed by residual tumour after completion of CRT (34%). Eight patients underwent primary surgical treatment due to contraindications to CRT, such as prior pelvic irradiation for another malignancy. An additional eight patients underwent surgery for post‐CRT sequelae, most commonly persistent perineal pain or non‐healing wounds despite a defunctioning colostomy.

**TABLE 2:** Details of surgeries performed for anal squamous cell carcinoma in Sweden 2017–2021 (*n* =103). Perioperative morbidity and mortality emerging within 30 days following surgical treatment are presented. Values are presented as *n* (%). Percentages are calculated within each surgical indication group based on its group total. The first column represents the entire patient cohort.

Indication for surgery		Recurrent tumour	Residual tumour	Primary surgical treatment	Re‐recurrence after salvage surgery	Sequelae
		44 (43)	35 (34)	8 (8)	8 (8)	8 (8)
Surgical procedures						
Abdominoperineal excision (APE)	60 (58)	25 (57)	24 (69)	7 (88)	–	4 (50)
Total pelvic exenteration (PE)	9 (9)	4 (9)	3 (9)	1 (13)	1 (13)	–
Posterior PE^a^	24 (23)	13 (30)	7 (20)	–	–	4 (50)
Anterior PE	1 (1)	1 (2)	–	–	–	–
Local excision	5 (5)	–	–	–	5 (63)	–
Inguinal lymphadenectomy only^b^	4 (4)	1 (2)	1 (3)	–	2 (25)	–
Abdominal surgical approach						
Open surgery	85 (83)	33 (75)	29 (83)	8 (100)	8 (100)	7 (88)
Laparoscopic surgery	16 (16)	10 (23)	5 (14)	–	–	1 (13)
Robot‐assisted surgery	2 (2)	1 (2)	1 (3)	–	–	–
Surgical reconstruction						
Flap reconstruction	91 (88)	38 (86)	34 (97)	7 (88)	4 (50)	8 (100)
Biological mesh reconstruction (perineal)	8 (8)	5 (11)	3 (9)	–	–	–
Overall morbidity (medical and/or surgical)	88 (85)	35 (80)	31 (89)	8 (100)	7 (88)	7 (88)
Surgical complications, any	81 (79)	34 (77)	28 (80)	8 (100)	6 (75)	5 (63)
Minor (CD I‐II)	79 (77)	33 (75)	28 (80)	8 (100)	5 (63)	5 (63)
Major	22 (21)	12 (27)	8 (23)	1 (13)	–	1 (13)
CD IIIa	9 (9)	6 (14)	3 (9)	–	–	–
CD IIIb	15 (15)	8 (18)	5 (14)	1 (13)	–	1 (13)
Mortality	0 (0)	–	–	–	–	–

*Note*: Complications are graded according to the Clavien‐Dindo (CD) classification.

Abbreviations: APE, abdominoperineal excision; PE, pelvic exenteration.

^a^ Female patients undergoing APE with or without hysterosalpingo‐oophorectomy.

^b^ One or several lymph nodes removed.

APE was the most common procedure (*n* = 60), followed by posterior pelvic exenteration (*n* = 24), total pelvic exenteration (*n* = 9), inguinal lymphadenectomy alone (*n* = 4) and anterior pelvic exenteration (*n* = 1). The latter was performed in a patient with a previous APE for inflammatory bowel disease; thus including a perineal excision but not a proctectomy. Six patients underwent a total of eight reoperations for confirmed re‐recurrence. Two of these patients had their initial salvage surgery before the study period, and two required reoperations twice during the study period. Re‐recurrence after previous salvage surgery (either abdominoperineal or nodal) was managed with local excision (*n* = 5), lymphadenectomy alone (*n* = 2) or total pelvic exenteration (*n* = 1).

APE was performed as an ELAPE in most patients (88/93; 95%). Multivisceral resection was performed in 74/99 (75%) procedures, including uni‐ or bilateral inguinal lymphadenectomies in 24 patients (Table S1). Flap reconstruction was carried out following 91 procedures, including 86 APE/extended APEs, one anterior pelvic exenteration, three local excisions and one lymph node dissection. The most used flap was the gluteal flap (*n* = 54), followed by the vertical rectus abdominis myocutaneous (VRAM) flap (*n* = 24), gracilis myocutaneous flap (*n* = 10) and composite flaps (*n* = 3). Composite reconstructions included: gluteal flap with vertical rectus abdominis myocutaneous flap and anterolateral thigh flap (n = 1), gluteal flap with lotus petal flap (*n* = 1) and VRAM combined with gracilis myocutaneous flap (*n* = 1).

### Surgical morbidity and mortality

The median operation time was 509 min (439–617), and intraoperative bleeding was 500 mL (200–1000). Postoperative complications, graded according to the Clavien‐Dindo classification, are summarised in Table 2. A detailed breakdown of all surgical complications occurring within 30 days postoperatively is provided in Table S2. Prolonged paralytic ileus (defined as requiring nasogastric tube and/or total parenteral nutrition) occurred in 43% of procedures. Transfusion‐requiring anaemia was observed in 48%. The median length of stay from admission to discharge from the operating unit was 20 days (13.5–27.5).

Surgical‐site infections were the most common postoperative complications, with approximately two‐thirds affecting the perineal wound, most managed conservatively with antibiotics. Perineal complications occurred after 47/98 (48%) resections, including perineal surgical‐site infections in 29/98 (30%) resections. Pelvic abscesses requiring ultrasound‐guided drainage were observed in seven patients. There were 16 unplanned reoperations, most commonly due to flap‐related complications (9/16; 56%), such as wound infection combined with flap necrosis and/or dehiscence. Other indications for reoperation included stomal revision (*n* = 2), intra‐abdominal bleeding (*n* = 1), pelvic bleeding (*n* = 1), bleeding gastric ulcer (*n* = 1), pelvic abscess (*n* = 1) and inguinal wound infection (*n* = 1). Perineal reconstruction methods and complications for patients undergoing abdominoperineal resection are summarised in Table 3.

**TABLE 3:** Perineal reconstruction and 30‐day surgical morbidity data in patients undergoing APE or extended APE for residual/recurrent ASCC or primary surgical treatment due to prior pelvic irradiation (*n* = 84). Values are presented as *n* (%), with percentages calculated within each reconstruction group. The first column represents the total cohort.

Flap type	Total (84)	Gluteal flap^a^ (45)	VRAM flap (20)	Gracilis flap (9)	Composite flaps (3)	Primary closure ± mesh (7)
Sex						
Female (*n*)	48 (57)	25 (56)	10 (50)	5 (56)	2 (67)	6 (86)
Surgical details						
Surgical complications, any	67 (80)	37 (82)	16 (80)	7 (78)	3 (100)	4 (57)
Minor (CD I–II)	66 (79)	37 (82)	16 (80)	7 (78)	3 (100)	3 (43)
Major	20 (24)	8 (18)	7 (35)	3 (33)	1 (33)	1 (14)
CD IIIa	8 (10)	3 (7)	2 (10)	2 (22)	0 (0)	1 (14)
CD IIIb	15 (18)	6 (13)	5 (25)	3 (33)	1 (33)	0 (0)
Perineal biological mesh	8 (10)	2 (4)	–	3 (33)	–	3 (43)
Vaginal reconstruction	29 (35)	15 (33)	9 (45)	3 (33)	1 (33)	1 (14)
Omental flap	15 (18)	11 (24)	1 (5)	–	–	3 (43)

*Note*: Complications are graded according to the Clavien‐Dindo (CD) classification.

Abbreviations: APE, abdominoperineal excision; ASCC, anal squamous cell carcinoma; VRAM, vertical rectus abdominis myocutaneous.

^a^ Gluteal V‐Y fasciocutaneous flap or gluteal myocutaneous flap.

Minor medical complications (Clavien‐Dindo I–II) occurred after 44 procedures (43%), while major medical complications (Clavien‐Dindo ≥ III) were observed in four patients. Recorded postoperative medical complications, including infectious, cardiovascular and neurological conditions, are detailed in Table S3. Most ICU admissions, corresponding to Clavien‐Dindo grade IV, resulted from perioperative conditions that do not clearly fit within the aforementioned medical categories and are therefore reported separately.

Unplanned ICU admissions occurred in 24 patients (25%). Indications for ICU admission, length of stay and associated outcomes are summarised in Table 4. Notably, two patients suffered cardiac arrest, one due to massive pulmonary aspiration and one due to electrolyte imbalance, both successfully resuscitated. No 30‐day mortality was observed, while one patient died within 90 days following withdrawal of ICU care.

**TABLE 4:** Summary of unplanned intensive care unit (ICU) admissions among all patients undergoing surgical treatment (*n* = 97), including indications, length of stay and outcomes.

Characteristics	*n*
Indications	
Circulatory instability with need of inotropic drugs	9
Respiratory insufficiency	6
Acute renal insufficiency	2
Refractory postoperative pain	3
Perioperative bleeding	2
Systemic inflammatory response syndrome	1
Metabolic seizures	1
Length of ICU stay, days	2 (IQR 1.8–5.3)
30‐day mortality	0
90‐day mortality	1

Abbreviations: ICU, intensive care unit; IQR, interquartile range.

### Risk factors for postoperative complications

The logistic regression analysis did not identify any statistically significant risk factors for major postoperative complications (Clavien‐Dindo ≥ III) within 30 days among patients undergoing APE or extended APE for oncological indications (Table 5).

**TABLE 5:** Univariate logistic regression analysis of risk factors affecting postoperative complications in ASCC patients undergoing APE or extended APE for residual/recurrent ASCC or primary surgical treatment due to previous pelvic irradiation (*n* = 84).

Characteristics	OR (95% CI)
Age	1.04 (0.99–1.09)
Gender	
Female	Reference
Male	1.00 (0.37–2.71)
ASA >1	
No	Reference
Yes	1.36 (0.14–12.86)
TNM at diagnosis	
T stage	
T1‐2	Reference
T3‐4	3.33 (0.87–12.81)
N stage	
N0	Reference
N+	1.23 (0.43–3.52)
M stage	
M0	Reference
M1	3.00 (0.18–50.33)
Perianal tumour component	
No	Reference
Yes	0.91 (0.33–2.51)
Indication for surgery^a^	
Residual tumour	Reference
Recurrent tumour	1.73 (0.60–4.98)
Time between radiation and surgery (months)	1.02 (0.98–1.06)
Total radiation dose	
≤58 Gy	Reference
≥58 Gy	2.40 (0.48–12.00)
Perineal reconstruction^b^	
Gluteal flap	Reference
VRAM flap	2.15 (0.67–6.97)
Surgical resection	
APE	Reference
Extended APE	2.27 (0.82–6.28)

*Note*: Missing data included T stage (*n* = 10, including Tx), *N* stage (*n* = 13), M stage (*n* = 6), tumour location (*n* = 5) and radiotherapy details (interval to surgery and total dose, *n* = 7). These cases were excluded from the regression analyses.

Abbreviations: ASCC, anal squamous cell carcinoma; APE, abdominoperineal excision; CRT, chemoradiotherapy; ASA, American Society of Anaesthesiologists; VRAM, vertical rectus abdominis myocutaneous.

^a^ Analysis was conducted exclusively on patients with residual or recurrent tumours (*n* = 76).

^b^ Analysis was limited to patients who received gluteal or VRAM flaps; those with other types of perineal reconstruction (*n* = 19) were excluded.

## DISCUSSION

This study, reporting outcomes for 97 patients treated at tertiary referral centres, focusing on 30‐day postoperative morbidity, presents the largest recent nationwide series of ASCC surgeries in Sweden. Most surgeries were performed for residual or recurrent disease following CRT. Perineal complications were the most common, though typically minor and managed conservatively. Still, 21% of patients required surgical or endoscopic intervention. Despite this, the 30‐ and 90‐day mortality was rates were low, 0 and 1%, respectively.

Previous studies on ASCC surgery have largely been limited to single‐centre case series [20]. In contrast, our study describes a cohort treated at specialised centres across Sweden, offering high external validity. The centralisation of ASCC surgery in 2017 aimed to ensure equitable care. Our observed rates of major complications, overall morbidity and perineal morbidity are comparable to those reported in previous studies (5–33%, 35–88% and 28–82%) following salvage surgery for residual or recurrent ASCC [21, 22, 23, 24, 25]. However, direct comparisons are difficult due to heterogeneous populations, surgical approaches, definitions of complications and follow‐up periods.

A Danish single‐centre study reported minor and major postoperative complications in 32% and 31% after APE with myocutaneous flap reconstruction and similarly found no clear predictors of major complications despite a sample size of 98 patients [26]. In our cohort, no significant associations were identified between major surgical complications and specific variables. Although we hypothesised that patients undergoing extended APE for recurrent disease would face higher complication risks due to radiation‐induced fibrosis, the trends observed for extended APE, recurrent tumours and primary tumour radiation dose of ≥58 Gy did not reach significance. Given the wide confidence intervals and limited sample size, these exploratory findings should be considered hypothesis‐generating and interpreted cautiously.

Major complications were more frequent with VRAM flaps (35%) than gluteal flaps (18%), though not statistically significant. This may reflect the greater technical complexity of VRAM reconstruction, often used for larger, irradiated defects in ASCC [27]. The predominance of gluteal flaps in our cohort likely suggests local preference and a desire to avoid abdominal donor‐site morbidity. Given the low‐quality evidence and absence of a clearly superior technique [27], high‐quality studies are needed to guide flap selection in irradiated perineal reconstructions.

Our results imply that surgery for ASCC is associated with considerable 30‐day postoperative morbidity. However, complications appear to be adequately identified and managed, reflected in the low 30‐ and 90‐day mortality following the centralisation of salvage surgery in Sweden. Despite this, there remains a need to improve management of the perineal wound after APE. The long‐term effects of perioperative perineal morbidity on wound healing, functional outcomes and quality of life warrant further investigation.

During the 5‐year study period (2017–2021), 97 patients underwent surgery for ASCC, representing approximately 10% of all ASCC patients in Sweden, based on an annual incidence of 200 ASCC cases. This confirms that surgery for ASCC remains uncommon, aligning with previously reported rates (15%) [28]. Compared to the broader national cohort of patients receiving radiotherapy, the surgical cohort in our study had a lower proportion of HPV‐positive (p16‐positive) tumours (71% vs. 90%), a lower median age (64 vs. 68 years) and fewer female patients (61% vs. 76%) [18, 29]. These differences reflect known prognostic factors, as male sex and HPV‐negative tumours are associated with a higher recurrence risk.

A key limitation of this study is its retrospective design, which relies on accurate electronic charting and registry data. Missing information and potential underreporting of minor complications may introduce information bias. We did not apply the Comprehensive complications index [30], as details for Clavien Dindo I–II complications can be difficult to elucidate from medical charts. Instead, we focused on major complications (Clavien‐Dindo ≥ III), which are more reliably recorded and clinically relevant.

Additionally, the study population was heterogeneous, including patients without preoperative CRT and those undergoing lymphadenectomy only. The heterogeneity in surgical indications, technique, extent of resection and flap reconstruction type influence complication risk and generalisability, thereby limiting interpretation of pooled complication rates across the cohort. To improve statistical homogeneity, only patients undergoing APE or extended APE for oncological indications (i.e. salvage for residual/recurrent tumours or primary surgery due to CRT contraindication) were included in statistical analysis. Although missing data were limited, using complete case analysis may have introduced bias; however, this is unlikely to have significantly impacted results. Together, these factors may have reduced the statistical power and limited the generalisability of the results.

A major strength of this study is its national, multicentre design, capturing all surgically treated ASCC patients in Sweden over a five‐year period. This enabled the inclusion of a relatively large and representative cohort despite the rarity of the disease. Additionally, the combination of meticulous medical chart review and national registry data enhances data reliability and reduces information bias through cross‐validation of clinical variables.

In conclusion, salvage surgery in Sweden is predominantly performed as ELAPE for residual or recurrent ASCC after initial CRT. While short‐term morbidity is substantial, with perineal complications occurring in nearly half of patients, most are managed conservatively. International multicentre collaborations with pooled data from specialised units will be essential to identify risk factors for perineal morbidity and establish a consensus on optimal perineal reconstructive strategies.

## AUTHOR CONTRIBUTIONS

**Stephanie Jacobsen:** Methodology; formal analysis; investigation; writing – original draft; visualization. **Eva Angenete:** Methodology; investigation; resources; writing – review and editing. **Anders Johnsson:** Methodology; investigation; writing – review and editing. **Marie‐Louise Lydrup:** Conceptualization; methodology; writing – review and editing. **Per J. Nilsson:** Methodology; investigation; resources; writing – review and editing. **Pamela Buchwald:** Conceptualization; methodology; writing – review and editing; project administration; funding acquisition.

## CONFLICT OF INTEREST STATEMENT

None declared.

## ETHICS STATEMENT

This study was approved by the Swedish Ethical Review Authority (reference number 2020–06085).

## Supporting information


Table S1.

## Data Availability

De‐identified individual participant data, including documentation detailing variable names and definitions, and the statistical analysis code is available from the corresponding author upon reasonable request.
