# Supplementary material for: Short‐term morbidity and mortality following surgical treatment of anal squamous cell carcinoma in Sweden – A national multicentre study
Source: Colorectal Dis. 2025 Oct 3;27(10):e70241. doi: 10.1111/codi.70241 (PMC12494122; doi:10.1111/codi.70241)

**Short-term morbidity and mortality following surgical treatment of anal squamous cell carcinoma in Sweden – a national multicentre study**

**Supplementary material.**

**Table S1.** **Extended resections**. Resection of additional organs and/or tissues beyond abdominoperineal resection, including all pelvic exenterations.

| Type of resection | Patients |
| --- | --- |
| Inguinal node dissection (uni-/bilateral) | 17/7 |
| Posterior vaginal wall^a^ | 38 |
| Other vaginectomy^b^ (partial or total) | 5 |
| Unilateral salpingo-oophorectomy (without hysterectomy) | 2 |
| Hysterectomy +/- salpingo-oophorectomy | 24 |
| Vulvectomy (partial^c^ or total) | 7 |
| Cystectomy | 10 |
| Ureterectomy (partial or total) | 3 |
| Urethrectomy (partial or total) | 5 |
| Prostatectomy (partial or total) | 6 |
| Seminal vesicles | 3 |
| Penectomy (partial (penile base) or total (incl. shaft)) | 6 |
| Orchiectomy | 2 |
| Pelvic bone^d^ (excl. coccyx) | 5 |
| Obturator nerve | 1 |
| Pelvic floor muscle^e^ (apart from m levator ani) | 6 |
| Pelvic hip muscles^f^ | 7 |
| Ileocecal resection/appendectomy | 4 |
| Mesenterectomy | 1 |
| Omentectomy | 2 |

^a^Including patients undergoing posterior exenteration (APE + hysterosalpingo-oophorectomy)

^b^lateral and/or anterior vaginal wall

^c^labium majus, labium minus, introitus.

^d^os sacrum, os pubis, os ischii

^e^m. coccygeus

^f^ m obturator internus, m piriformis, mm. glutei

| Surgical complications | n of events | n of patients | Minor complications (CD 2) | Major complications  (CD ≥ 3) | Comment to CD ≥ 3 |
| --- | --- | --- | --- | --- | --- |
| Abdominal | | | | | |
| Wound infection | 13 | 13 | 10 | 3 | revision and VAC (n=1), reoperation (n=1), wound revision with concurrent drainage of abscess (n=1) |
| Abscess | 4 | 4 | 3 | 1 | surgical drainage (n=1) |
| Postoperative bleeding | 7 | 7 | 4 | 3 | reoperation (n=2), superficial wound bleed (n=1) |
| Stomal complication | 3 | 3 | 1 | 2 | reoperation with revision (n=2) |
| Other (bleeding gastric ulcer, ileus, seroma, abdominal wall hernia, donor site necrosis) | 4 | 4 | 3 | 1 | gastro- and coloscopy (n=1) |
| Perineal |  |  |  |  |  |
| Wound infections | 29 | 29 | 24 | 5 | surgical revision (n=2), reoperation with new flap (n=2), revision and drainage of concurrent abscess (n=1) |
| Abscess | 14 | 14 | 6 | 8 | percutaneous drainage (n=7), drainage under anaesthesia during perineal revision (n=1) |
| Fascial dehiscence | 11 | 11 | 3 | 8 | revision under anaesthesia (n=6), new flap (n=2) |
| Flap necrosis | 6 | 6 | 0 | 6 | removal of flap (n=1), new flap (n=3), revision (n=2) |
| Wound revisions due to perineal complications | 37 | 11 |  |  |  |
| Vacuum assisted closure |  | 12 |  |  |  |

**Table S2.** Surgical complications. Surgical complications (n=81 patients in total). Multiple complications were registered for some patients.

**Table S3**. 30-day postoperative medical complications. Postoperative complications graded according to the Clavien-Dindo Classification. Multiple complications were registered for some patients, why the total number of complications exceeds the number of patients operated.

| Complication | n of patients | n of events | Minor complications  (CD 2) | Major complications  (CD ≥ 3) | Comment to CD ≥ 3 |
| --- | --- | --- | --- | --- | --- |
| Infectious | 38 | 44 |  |  |  |
| Sepsis |  | 6 | 6 | 0 |  |
| Pneumonia |  | 10 | 8 | 2 | Intensive care due to respiratory failure (n=2) |
| Other infectious complications |  | 28 | 28 | 0 | UTI (n=12), infection of unknown origin (n=11), oral candida (n=5) |
| Cardiovascular | 14 | 18 |  |  |  |
| Myocardial infarction |  | 1 | 1 | 0 |  |
| Heart failure |  | 2 | 2 | 0 |  |
| Arrythmia |  | 9 | 8 | 1 | Pacemaker insertion(n=1) |
| Pulmonary embolism |  | 5 | 4 | 1 | Inferior vena cava filter placement due to concurrent bleeding (n=1) |
| Deep vein thrombosis |  | 1 | 1 | 0 |  |
| Neurological | 6 | 6 |  |  |  |
| Confusion |  | 5 | 5 | 0 |  |
| Limb weakness |  | 1 | 1 | 0 |  |

**Fig S1.** This Directed Acyclic Graph (DAG) illustrates the assumed causal relationships among variables and influenced the selection of covariates for the logistic regression analysis to appropriately adjust for confounding factors.
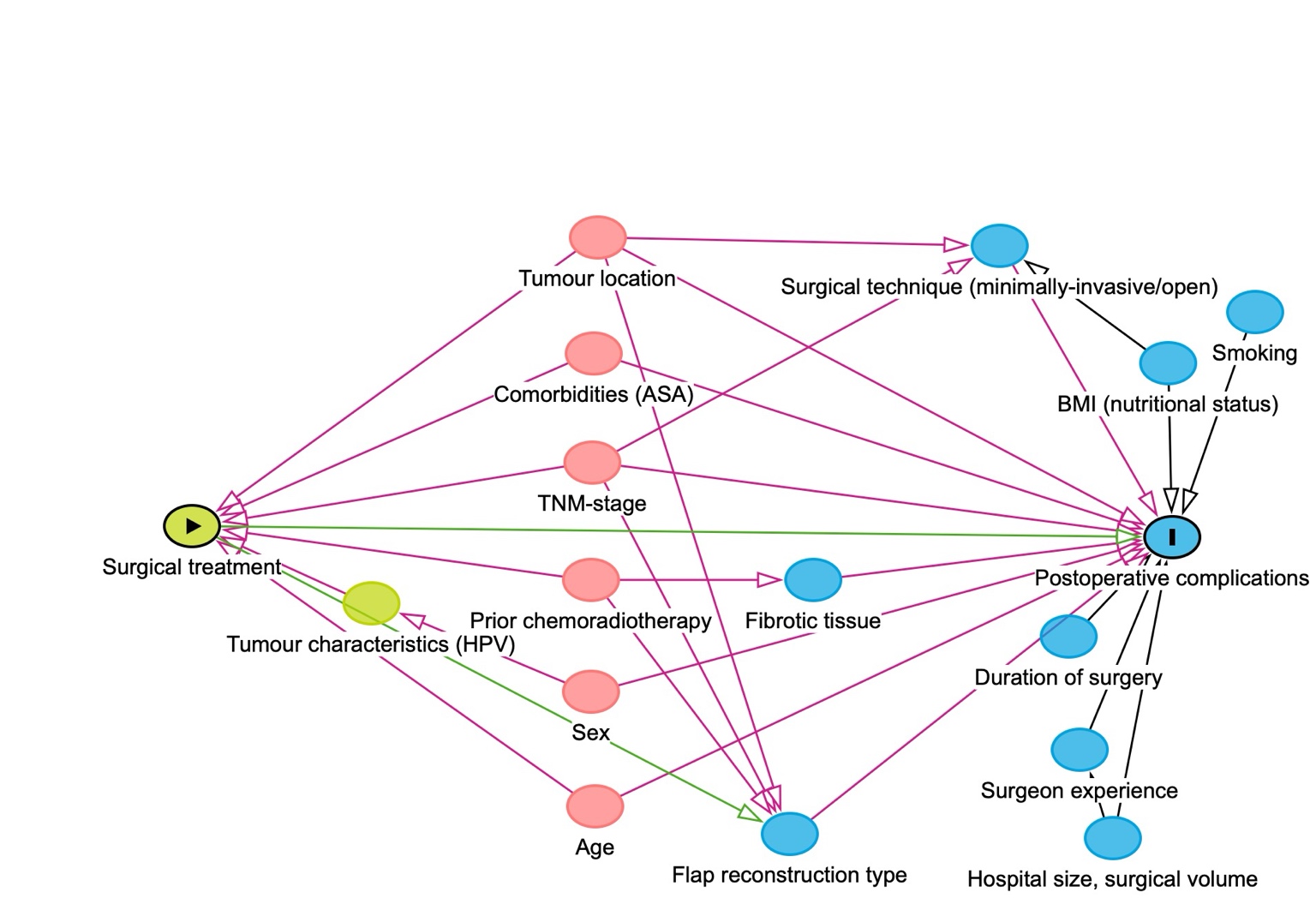

Supplement: Supplementary file 1 — Table S1. [file CODI-27-0-s001.docx]
